# In this month’s Bulletin

**DOI:** 10.2471/BLT.20.000120

**Published:** 2020-01-01

In the editorial section this month, Shamsuzzoha Babar Syed et al. (2) ask how quality of care can be improved in fragile, conflict-affected and vulnerable settings. The World Health Organization (3) issues a retraction statement on guidance for opioid use. 

In the news section Sophie Cousins (6–7) reports on health service outreach using hospital trains in China and India. Gary Humphreys interviews Kathryn Chu (8–9) on the intersection of surgery and public health and her research on improving the quality of surgical delivery in humanitarian crises. 

## Bangladesh, India

### Essential health interventions

Daphne CN Wu et al. (19–29) review investments made for urban populations.

## China

### Blood-banking in 2036

Xiaochu Yu et al. (10–18) project per-capita blood supply and demand.

## Germany

### Hot and cold spots for asthma 

Manas K Akmatov et al. (40–51) analyse the spatial distribution of outpatient claims for asthma treatment.

## Ghana, Kenya, Liberia, Malawi, Nigeria, Rwanda, Uganda, United Republic of Tanzania, Zambia, Zimbabwe

### Monitoring sustainable development

Andrea Farnham et al. (69–71) argue for more use of district health information.

## Global

### Supporting community health workers 

Chunling Lu et al. (30–39) estimate amounts of development assistance provided to 114 countries.

### Ending involuntary treatment 

Kanna Sugiura et al. (52–58) examine the evolution of mental health care.

### Affordable measurement of quality-adjusted life years

Yot Teerawattananon et al. (59–65) discuss charging policies for survey instruments.

### Reducing unnecessary caesarean sections

Newton Opiyo et al. (66–68) present WHO recommendations for non-clinical interventions.

